# Complete Genome Sequence of a Boa (*Boa constrictor*)-Specific Papillomavirus Type 1 Isolate

**DOI:** 10.1128/MRA.01159-18

**Published:** 2018-10-25

**Authors:** Jakub Kubacki, Anna Sophie Ramsauer, Claudia Bachofen, Claude Favrot, Alexandra Nicolier, Cornel Fraefel, Kurt Tobler

**Affiliations:** a Institute of Virology, Vetsuisse Faculty, University of Zurich, Zurich, Switzerland; b Dermatology Unit, Vetsuisse Faculty, University of Zurich, Zurich, Switzerland; c Vet Diagnostics, Lyon, France; KU Leuven

## Abstract

We present the full-length genome sequence of a new papillomavirus detected in skin lesions collected from a boa (Boa constrictor). Based on the nucleotide sequence analysis, we propose to designate the newly identified virus as Boa constrictor *papillomavirus type 1* (BcPV1), a new species in the genus Dyomupapillomavirus.

## ANNOUNCEMENT

Papillomaviruses (PVs) are nonenveloped circular double-stranded DNA viruses and are among the most widespread animal viruses (1). The *Papillomaviridae* family contains more than 320 PV types. Most have been identified as infecting skin and mucosal epithelium in mammalian hosts. To our knowledge, only nine nonmammalian PVs have been described, from birds, turtles, fish, and a snake (2). Previously, the only known snake PV, which was detected in a diamond python (Morelia spilota spilota) and named MsPV1, represented the first complete PV genome discovered in a *Squamata* host. Interestingly, MsPV1 does not cluster with any other nonmammalian PVs and has been the only PV in the Dyomupapillomavirus genus (3, 4).

Here, we present the full-genome sequence of a second snake papillomavirus, detected in skin lesions from a 5-year-old female boa (Boa constrictor) from the zoo in La Barben, France. The snake revealed several small hyperkeratotic plaques located in the first third of the animal without any evidence of discomfort. DNA was extracted from a biopsy specimen of one of these skin lesions with the QIAamp DNA minikit according to the manufacturer’s protocol (Qiagen GmbH, Germany). Initially, PCR with a degenerate consensus primer (FAP59/64) for the L1 gene was used to amplify potential PV genome fragments (5). Sequencing of the resulting approximately 480-bp PCR product yielded a putatively novel PV DNA sequence. Hence, circular viral DNA was amplified by rolling circle amplification (RCA), with a TempliPhi amplification kit (GE Healthcare Lifesciences), and then digested with BamHI and NarI restriction endonucleases and gel purified with a Zymoclean gel DNA recovery kit (Zymo Research).

To prepare the sample for next-generation sequencing (NGS), a sequencing library was prepared from 1 ng of DNA with the NEBNext Ultra II DNA library preparation kit and the NEBNext Multiplex Oligos for Illumina barcoding kit according to the manufacturer’s instructions (New England Biolabs, Switzerland). A paired-end NGS run of 2 × 150-nucleotide (nt) read length, using the high-output flow cell, was performed on an Illumina NextSeq 500 sequencing system at the Functional Genomics Center Zurich in Zurich, Switzerland. *De novo* assembly of 14.3 million generated reads with SeqMan NGen software (Lasergene v.15; DNASTAR, Madison, WI) revealed 8.3 million reads that formed a single consensus sequence of a papillomavirus. Alignment of the consensus sequence to papillomavirus genomes showed that it is a new papillomavirus, genetically most closely related (54% identity) to MsPV1 (3). The L1 open reading frame (ORF) shares 65.9% nucleotide sequence similarity with L1 of MsPV1. With this level of sequence identity, the new virus may be considered a new species within the genus Dyomupapillomavirus (6). The 7,126-nt full-length *Boa constrictor* papillomavirus type 1 (BcPV1) (isolate CH_2018_UZH) sequence with a GC content of 40.3% contains the typical PV ORFs, namely, E6, E7, E1, E2, E4, L1, and L2, which are specified in the GenBank entry.

In conclusion, we detected a new snake PV (BcPV1) in skin lesions from a boa in the zoo in La Barben, France, using NGS. BcPV1 forms a new species in the genus Dyomupapillomavirus.

### Data availability.

This genome sequence is deposited in GenBank under the accession number MH605022.
